# The miR-184 Binding-Site rs8126 T>C Polymorphism in *TNFAIP2* Is Associated with Risk of Gastric Cancer

**DOI:** 10.1371/journal.pone.0064973

**Published:** 2013-05-28

**Authors:** Yu Xu, Hongxia Ma, Hongping Yu, Zhensheng Liu, Li-E Wang, Dongfeng Tan, Ramya Muddasani, Victoria Lu, Jaffer A. Ajani, Yanong Wang, Qingyi Wei

**Affiliations:** 1 Department of Epidemiology, The University of Texas M.D. Anderson Cancer Center, Houston, Texas, United States of America; 2 Department of Pathology, The University of Texas M.D. Anderson Cancer Center, Houston, Texas, United States of America; 3 Department of GI Medical Oncology, The University of Texas M.D. Anderson Cancer Center, Houston, Texas, United States of America; 4 Department of Gastric Cancer and Soft Tissue Sarcomas Surgery, Fudan University Shanghai Cancer Center, Shanghai, China; University of Illinois at Chicago, United States of America

## Abstract

**Background:**

*TNFAIP2* is a crucial gene involved in apoptosis. Single nucleotide polymorphisms (SNPs) in its miRNA binding sites could modulate functions of the miRNA-target genes and thus risk of cancers. In this study, we investigated associations between potentially functional SNPs in the miRNA binding sites of the 3′UTR of *TNFAIP2* and gastric cancer risk in a US population.

**Methods:**

We conducted a case-control study of 301 gastric cancer patients and 313 cancer-free controls frequency-matched by age, sex and ethnicity. We genotyped four selected *TNFAIP2* SNPs (rs8126 T>C, rs710100 G>A, rs1052912 G>A and rs1052823 G>T) and used the logistic regression analysis to assess associations of these SNPs with cancer risk.

**Results:**

The rs8126 CC genotype was associated with a significantly elevated risk of gastric cancer (adjusted OR = 2.00, 95% CI = 1.09–3.64 and *P* = 0.024), compared with the combined rs8126 TT+TC genotypes, particularly in current drinkers. However, none of other *TNFAIP2* SNPs was associated with risk of gastric cancer.

**Conclusions:**

Our data suggested that the *TNFAIP2* miRNA binding site rs8126 T>C SNP may be a marker for susceptibility to gastric cancer, and this finding requires further validation by larger studies.

## Introduction

Gastric cancer is one of the major causes of cancer-related deaths in the world, although both its incidence and mortality have been declining in the latest decade [1]. In 2010, there were approximately 21,000 newly diagnosed patients and 10,570 deaths of this disease in the United States [2]. Epidemiological studies have suggested that environmental factors, including diet high in salted and nitrated foods, tobacco use, alcoholic consumptions and, especially, *Helicobacter pylori* (*H. pylori*) infection, are important factors for the etiology of gastric cancer [3]. However, only a fraction of people adopting similar life styles or exposed to the same environmental risk factors eventually developed gastric cancer, suggesting that host or genetic factors may also play a role in the etiology of the disease [4].

Among all pathophysiologic changes caused by various etiologic factors related to gastric cancers, the induction of oxidative DNA damage in epithelial cells of the stomach has been demonstrated to be a strong link to carcinogenesis [5], [6]. To prevent an uncontrolled cell growth resulting from this kind of DNA damage, some cell cycle control mechanisms must be initiated to prevent the occurrence of mutations or to initiate apoptosis of the cells with overwhelming damage to the DNA.

MicroRNAs (miRNAs), a class of endogenous and small non-coding regulatory RNA, negatively regulate gene expression at the post-transcriptional level through hybridization to complementary sequences in the 3′ untranslated region (3′UTR) of the target messenger RNAs (mRNAs) [7], [8], [9], [10]. Recent studies have shown significant effects of miRNAs on various biological processes, including cell differentiation, proliferation, cell cycle progression as well as apoptosis. Altered expressions of miRNAs as well as their potential functions of tumor suppressors and oncogenes have been demonstrated in several types of cancer, including gastric cancer [11], [12]. Meanwhile, several relatively common genetic variants, such as single nucleotide polymorphisms (SNPs), have been identified as biomarkers for genetic susceptibility to cancer by molecular epidemiological studies. Due to their potential effects on miRNA expression and subsequent impact on mRNA transcription, miRNA SNPs or miRNA-related SNPs, such as those located in the miRNA binding sites at the 3′ untranslated region (UTR) of their target genes, have been implicated as crucial genetic factors in susceptibility to various cancers. Since their roles in cancer etiology remains unclear, it is important to understand functional and evolutionary significance of these miRNA-related SNPs through their effects on cancer risk and their interaction with environmental risk factors in the etiology of gastric cancer [13].

**Figure 1:**
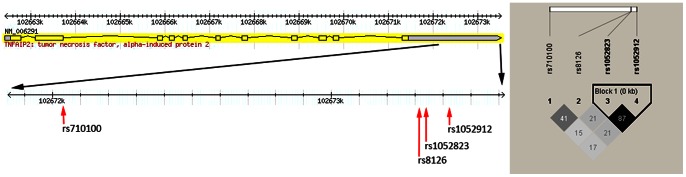
The four selected SNPs of *TNFAIP2*. (A) The four validated SNPs and their exact locations within the *TNFAIP2* 3′-UTR. (B) Linkage disequilibrium (LD) map for selected SNPs of *TNFAIP2* gene. It showed that rs1052912 and rs1052823 are in LD with a D′ = 0.87.

Belonging to the SEC6 family, the tumor necrosis factor α-induced protein 2 (TNFAIP2, also known as B94 or EXOC3L3), which is one kind of pro-apoptotic protein, was originally identified as a gene whose expression can be induced by the tumor necrosis factor alpha (TNFα) in umbilical vein endothelial cells [14]. *TNFAIP2* is located on chromosome 14q32 and encodes a protein of 654 amino acid residues with an apparent molecular weight of 73 kDa. Consisting of 11 exons and 10 introns, *TNFAIP2* spans approximately 13.45 kb of genomic DNA [15]. In the dbSNP database (http://egp.gs.washington.edu/), this gene is reported to have 180 SNPs, of which 15 SNPs in the 3′UTR of *TNFAIP2* are located in the predicted miRNAs binding sites, but only four of these miRNA-related SNPs are common [i.e. minor allele frequency (MAF)>0.05]. These four SNPs are rs8126 T>C (localized within the binding site for miR-184), rs710100 G>A (within miR-155), rs1052912 G>A (within miR-105) and rs1052823 G>T (within miR-550) (http://compbio.uthsc.edu/miRSNP) [**Fig. 1A**].

Recent studies have shown that these SNPs in the miRNA binding sites may potentially be associated with human cancer risk [15], [16]. Therefore, we hypothesized that *TNFAIP2* SNPs are associated with susceptibility to gastric cancer. To test this hypothesis, we conducted a case-control study to evaluate the association between these four *TNFAIP2* SNPs in miRNA binding sites and risk of gastric cancer in a US non-Hispanic white population.

## Materials and Methods

### Study Subjects

Study subjects were patients with newly diagnosed and histologically confirmed gastric cancer at The University of Texas M.D. Anderson Cancer Center (Houston TX) between March 2002 and February 2011, who were recruited for an ongoing case-control study of gastric cancer. At the same time, cancer-free control subjects were recruited by using frequency matching on age (±5 years), sex, and ethnicity (non-Hispanic whites vs. others) from an ongoing molecular epidemiology study of head and neck cancer between 2002 and 2011 [17]. These cancer-free control subjects were not hospital patients who were seeking health care, nor genetically unrelated to the cases. Additional information about risk factors, such as smoking and alcohol use, was collected from each eligible subject who had provided informed consent. The study protocol was approved by The University of Texas M.D. Anderson Cancer Center institutional review board.

### Genotyping

Genomic DNA was extracted from the buffy coat fraction of each blood sample by using a Blood Mini Kit (Qiagen, Valencia, CA) according to the manufacturer's instructions. DNA purity and concentrations were determined by spectrophotometric measurement of absorbance at 260 and 280 nm by UV spectrophotometer.

The selected two *TNFAIP2* SNPs, rs710100 and rs1052912, were genotyped by using the TaqMan methodology in 384-well plates and read with the Sequence Detection Software on an ABI-Prism 7900 instrument, according to the manufacturer's instructions, Applied Biosystems (Foster City, CA), who also supplied primers and probes. Each plate included four negative controls, duplicated positive controls and eight repeated samples. The conditions of amplification were as follows: 50°C for 2 min, 95°C for 10 min followed by 40 cycles of 95°C for 15 sec, and 60°C for 1 min.

Because the TaqMan assay was not suitable for the other two SNPs, i.e., rs8126 and rs1052823, they were genotyped by the PCR-restriction fragment length polymorphism method, in which the primers were designed to create new restriction sites and used to amplify the fragments that contain the polymorphisms for the both two SNPs.[18] The sequences of the primers used for genotyping assays were 5′-GGGGCCGGCTCTCTTGGGCC-3′ and 5′-CACACGTACAAAGACCTTGGGCATCC-3′ for rs8126, and 5′-CCTTCGGACTCAGGCATGACTC-3′ and 5′-GTAAGGCAGTACCTGGGAAAAGGGTA-3′ for rs1052823. The PCR profile consisted of an initial melting step of 95°C for 5 min, 35 cycles of 95°C for 30 s, 60°C for 45 s and 72°C for 1 min and a final extension step of 72°C for 10 min. The PCR products for rs8126 C allele of 105 base pairs (bp) were digested by the *Apa*I enzyme (New England Biolabs, Beverly, MA) to two fragments of 85 bp and 20 bp, while those of rs1052823 G allele of 108 bp were digested by the *Rsa*I (New England Biolabs) to three fragments of 83 bp, 15 bp and 10 bp. The assay success rate for all genotypes was >99%, and the repeated assays for >10% of the samples were 100 concordant.

### Statistical Analysis

The χ^2^ tests were performed to compare the distribution of demographic variables and selected risk factors, such as smoking and alcoholic consumptions, between cases and controls. The Hardy-Weinberg equilibrium was tested by a goodness-of-fit χ^2^ test to compare the observed genotype frequencies with the expected ones in cancer-free controls. The associations of genotypes of *TNFAIP2* SNPs with risk of gastric cancer were estimated by calculating the odds ratios (OR) and their 95% confidence intervals (CIs) from both univiariate and multivariate logistic regression models in case-control analyses, followed by stratification analysis by age (≤59 vs.>59 years), sex, ethnicity (white vs. non-white), smoking and drinking status. All these analyses were performed with or without adjustment for demographic variables and selected risk factors. Proc HAPLOTYPE in SAS/Genetics software using the expectation-max-imization (EM) algorithm was conducted to generate maximum likelihood estimates of haplotype frequencies. Haplotypes with frequencies <5% were combined into one group. All tests were two-sided, and a *P*<0.05 was considered the cutoff for statistical significance. All of the statistical analyses were performed with Statistical Analysis System software (Version 9.2; SAS Institute, Cary, NC).

## Results

### Demographic Characteristics and Risk Factors for Gastric Cancer

This study included 301 gastric cancer patients and 313 cancer-free controls. **Table 1** summarizes the distribution of demographic characteristics and selected risk factors for gastric cancer. Because of frequency matching used in our study design, there were no significant difference in distributions of age (median age of 59 yrs), sex and ethnicity. Apparently, there was no difference in smoking and drinking status between cases and controls. However, these variables were further adjusted for in further multivariate logistic regression models to control for any residual confounding on the main effect of selected SNPs.

**Table 1:** Distributions of selected variables in gastric cancer cases and cancer-free controls.

Variables	Cases No. (%)	Controls No. (%)	*P* a
All subjects	301 (100%)	313 (100%)	
Age, yr (mean±SD)	59.45±12.28	59.42±11.88	0.517
≤59 (median)	146 (48.50%)	160 (51.12%)	
>59 (median)	155 (51.50%)	153 (48.88%)	
Sex			0.488
Males	109 (36.21%)	105 (33.55%)	
Females	192 (63.79%)	208 (66.45%)	
Ethnicity			0.858
White	197 (65.45%)	207 (66.13%)	
Non-white	104 (34.55%)	106 (33.87%)	
Smoking Status			0.473
Never	140 (46.51%)	161 (51.44%)	
Former	114 (37.87%)	107 (34.19%)	
Current	47 (15.61%)	45 (14.38%)	
Drinking Status^b^			0.372
Never	146 (48.99%)	142 (45.37%)	
Former	49 (16.44%)	65 (20.77%)	
Current	103 (34.56%)	106 (33.87%)	

a Two-sided χ^2^ test.

b Information of smoking and drinking status was unavailable for 3 cases.

### *TNFAIP2* Genotypes and Risk of Gastric Cancer

The genotype distributions of the selected four *TNFAIP2* SNPs between the cases and controls are listed in **Table 2**. The observed genotype frequencies of these SNPs were all in agreement with those expected by the Hardy-Weinberg equilibrium in the subjects (*P* = 0.234 for rs1052823 G>T, *P* = 0.698 for rs710100 G>A and *P* = 0.125 for rs1052912 G>A), except for rs8126 T>C (*P* = 0.013) in the cases. The distributions of the genotypes for *TNFAIP2* rs8126 T>C were 56.48% for TT, 32.56% for CT and 10.96% for CC in the cases, significantly different from those in the controls, which was 52.72% for TT, 41.53 for CT and 5.75 for CC. This difference was statistically significant (*P* = 0.019) under the recessive model (rs8126 T>C CC/TT+CT). (**Table 2**)

**Table 2:** Logistic regression analysis of associations between the genotypes of *TNFAIP2* and gastric cancer risk.

Genotypes	Cases		Controls		*P*	Crude OR (95% CI)	Adjusted OR^a^ (95% CI)	*P* a
	n	%	n	%				
*TNFAIP2* rs8126 T>C					**0.013**			
TT	170	56.48	165	52.72		1.00	1.00	
CT	98	32.56	130	41.53		0.73 (0.52–1.03)	0.74 (0.53–1.05)	0.088
CC	33	10.96	18	5.75		1.78 (0.96–3.28)	1.76 (0.95–3.27)	0.072
Dominant Model	CT+CC vs. TT				0.350	0.86 (0.63–1.18)	0.87 (0.63–1.20)	0.383
Recessive Model	CC vs. TT+CT				**0.019**	**2.02 (1.11–3.67)**	**2.00 (1.09–3.64)**	**0.024**
*TNFAIP2* rs1052823 G>T					0.234			
GG	246	81.73	239	76.36		1.00	1.00	
GT	50	16.61	69	22.04		0.70 (0.47–1.06)	0.71 (0.47–1.07)	0.101
TT	5	1.66	5	1.60		0.97 (0.28–3.40)	0.92 (0.26–3.26)	0.899
Dominant Model	GT+TT vs. GG				0.103	0.72 (0.49–1.07)	0.73 (0.49–1.08)	0.111
Recessive Model	TT vs. GG+GT				0.950	1.04 (0.30–3.63)	0.99 (0.28–3.49)	0.984
*TNFAIP2* rs710100 G>A					0.698			
GG	120	39.87	135	21.99		1.00	1.00	
AG	141	46.84	137	43.77		1.16 (0.82–1.63)	1.18 (0.83–1.66)	0.356
AA	40	13.29	41	13.10		1.10 (0.67–1.81)	1.08 (0.65–1.79)	0.781
Dominant Model	AG+AA vs. GG				0.412	1.14 (0.83–1.58)	1.15 (0.83–1.60)	0.392
Recessive Model	AA vs. GG+AG				0.945	1.02 (0.64–1.62)	0.99 (0.61–1.59)	0.956
*TNFAIP2* rs 1052912 G>A					0.125			
GG	249	82.72	240	76.68		1.00	1.00	
AG	52	17.28	72	23.00		0.70 (0.47–1.04)	0.70 (0.47–1.04)	0.080
AA	0	0	1	0.32		NA	NA	0.986
Dominant Model	AG+AA vs. GG				0.063	0.69 (0.46–1.03)	0.69 (0.46–1.03)	0.069
Recessive Model	AA vs. GG+AG				0.326	NA	NA	0.986

a Adjusted for age, sex, race, smoking status and drinking status.

(Statistically significant findings are in bold).

Additionally, the *TNFAIP2* SNP rs8126 T>C, the only one out of the four selected *TNFAIP2* SNPs in miRNA binding sites, showed a statistically significant association in further logistic regression analysis. Specifically, the rs8126 homozygous CC genotype was only borderline associated with an increased risk of gastric cancer (adjusted OR = 1.76, 95% CI = 0.96–3.27 and *P* = 0.072), compared with the TT genotype, but this risk increased (adjusted OR = 2.00, 95% CI = 1.09–3.64 and *P* = 0.024), when compared to the combined rs8126 T variant genotypes (**Table 2**). However, in the following stratified analysis by age, sex, ethnicity, smoking and drinking status, only in the subgroup of current smokers, but not drinkers, did the elevated risk remain statistically significant with a larger variation in the estimates (adjusted OR = 4.04, 95% CI = 1.08–15.08 and *P* = 0.038) (**Table 3**), although the subgroup had limited observations. No other significant association was found in the analysis for joint effects of all four *TNFAIP2* SNPs in the miRNA binding sites.

**Table 3:** Stratification analysis for associations between the *TNFAIP2* rs8126 SNP and gastric cancer risk.

Variables	rs8126 T>C (cases/controls)		Crude OR (95% CI)	Adjusted OR^a^ (95% CI)	*P* a
	TT+CT	CC			
Age, yr					
≤59 (median)	133/154	13/6	2.51 (0.93–6.78)	2.51 (0.92–6.83)	0.073
>59 (median)	135/141	20/12	1.74 (0.82–3.70)	1.76 (0.82–3.76)	0.146
Sex					
Males	95/97	14/8	1.79 (0.72–4.46)	1.94 (0.76–4.98)	0.169
Females	173/198	19/10	2.17 (0.98–4.80)	2.08 (0.94–4.61)	0.073
Ethnicity					
White	177/195	20/12	1.84 (0.87–3.87)	1.85 (0.87–3.91)	0.108
Non-white	91/100	13/6	2.38 (0.87–6.52)	2.30 (0.84–6.35)	0.107
Smoking Status					
Never	125/152	15/9	2.03 (0.86–4.79)	1.93 (0.80–4.67)	0.146
Former	102/101	12/6	1.98 (0.72–5.48)	1.95 (0.70–5.43)	0.202
Current	42/41	6/3	2.05 (0.48–8.74)	2.11 (0.49–9.17)	0.318
Drinking Status^b^					
Never	127/132	19/10	1.98 (0.88–4.41)	1.98 (0.88–4.47)	0.101
Former	46/60	3/5	0.78 (0.18–3.45)	0.83 (0.18–3.79)	0.809
Current	92/103	11/3	**4.11 (1.11–15.17)**	**4.04 (1.08–15.08)**	**0.038**

a Adjusted for age, sex, race, smoking status and drinking status.

b Information of drinking status was unavailable for 3 cases.

(Statistically significant findings are in bold).

### *TNFAIP2* Haplotypes and Risk of Gastric Cancer

The haplotypes were also explored to determine whether any particular haplotype may be associated with gastric cancer risk. As shown in **Fig. 1B**, *TNFAIP2* rs1052823 and rs1052912 are in high linkage disequilibrium (LD) (r^2^ = 0.87); consequently, rs1052912 was not included in the haplotype analysis. Four haplotypes were shown to have frequencies >5% among all the cases, while other less common haplotypes (frequencies <5%) were combined into one group in the analysis. The four common haplotypes (rs8126/rs1052823/rs710100: T-G-G, C-G-A, T-G-A and C-T-A) accounted for 90.53% and 94.72% of the chromosomes of the cases and controls, respectively. However, the frequency distribution of haplotypes was not statistically different between cases and controls, and their associations with risk of gastric cancer were not significant as well. In further analysis on the other uncommon haplotypes, the haplotype rs8126/rs1052823/rs710100: C-G-G showed a statistically significant association with cancer risk (adjusted OR = 2.60, 95% CI = 1.39–4.85 and *P* = 0.003), compared with the common haplotype T-G-G. (**Table 4**)

**Table 4:** Haplotype analysis for genotypes of *TNFAIP2* and Gastric Cancer risk.

Haplotypes a	Haplotype frequencies				Crude OR (95% CI)	Adjusted OR^a^ (95% CI)	*P* a
	Cases		Controls				
	n	%	n	%			
T-G-G	341	56.64	383	61.18	1.00	1.00	
C-G-A	88	14.62	85	13.58	1.16 (0.83–1.62)	1.17 (0.84–1.63)	0.365
T-G-A	78	12.96	64	10.22	1.37 (0.95–1.97)	1.35 (0.94–1.95)	0.110
C-T-A	38	6.31	61	9.74	0.70 (0.46–1.08)	0.71 (0.46–1.09)	0.117
**Others**	**57**	**9.47**	**33**	**5.27**	**1.18 (1.05–1.32)**	**1.18 (1.05–1.32)**	**0.005**
C-G-G	35	5.81	15	2.40	**2.62 (1.41–4.88)**	**2.60 (1.39–4.85)**	**0.003**
T-T-A	17	2.82	9	1.44	2.12 (0.93–4.82)	2.10 (0.92–4.80)	0.077
C-T-G	3	0.50	5	0.80	0.67 (0.16–2.84)	0.64 (0.15–2.70)	0.539
T-T-G	2	0.33	4	0.64	0.56 (0.10–3.09)	0.53 (0.10–2.95)	0.471

a The order of haplotype sequence is rs8126, rs1052823 and rs710100.

Adjusted for age, sex, race, smoking status and drinking status.

(Statistically significant findings are in bold).

## Discussion

In this hospital-based case-control study, we found that the *TNFAIP2* miR-184 binding site variant rs8126 CC genotype was significantly associated with an elevated risk of developing gastric cancer in a recessive genetic model. In stratification analysis, such an effect was more evident in the subgroup of current drinkers, which could be a chance finding. However, there was no statistical evidence of an association with risk of the disease for variant genotypes of other selected SNPs, including rs1052823 G>T, rs710100 G>A and rs1052912 G>A, either in overall analyses or in stratified analyses by age, sex, ethnicity, smoking status or drinking status. Moreover, the haplotype analysis did not identify any additional subgroups with common frequency at high risk.

Recently, two genome-wide association studies (GWASs) have reported significant associations of several genetic variants with gastric cancer risk in Chinese populations as well as Japanese and Korean populations [19], [20]. SNPs of the *TNFAIP2* gene were not among the reported top-hits, because only allelic associations rather than genetic models, such as the recessive model, were tested in these GWAS studies. In the present study with a limited sample size, the selected SNP rs8126 T>C SNP in the *TNFAIP2* miRNA binding site, a SNP that was not included, nor in LD with those included, in the GWAS chip, was associated with gastric cancer. This finding, however, needs further validation by larger studies.

Few studies have investigated the role of the SNPs in the miRNA binding sites in the etiology of gastric cancer. A case-control association study in a Japanese population of 552 cases and 697 controls demonstrated that the rs2910164 CC genotype in pre-miRNAs (miR-146a) was statistically associated with an increased risk of gastric cancer [21]. Another similar gastric cancer study in a Chinese population demonstrated a significantly increased risk in subjects with the variant CC homozygotes of miR-196a-2 compared with the wild-type homozygous TT and heterozygous CT carriers. This SNP was also shown to have a strong association with lymph node metastasis [22]. Similar effects were found for the combined rs895819 AG+GG genotypes of hrs-mir-27a in a Chinese study carried out by Qingmin S *et al* [23]. Further functional analyses indicated that the variant C allele might be responsible for elevated expression of miR-27a, by reducing mRNA expression of its target gene, *ZBTB10* (Zinc finger and BTB domain containing 10), a possible mechanism by which the *hsa-mir-27a* SNP plays a role in gastric cancer susceptibility [23]. However, no published studies have investigated the role of SNPs residing in the miRNA binding sequence in gastric cancer.

*TNFAIP2* (*B94*) is a cytokine-driven primary response gene that was originally cloned from TNF-α-inducible transcript in human stimulated endothelial cells [15]. Further studies found that it could be activated by factors other than TNF including interleukin-1β or lipopolysaccharide [24]. The expression of *TNFAIP2* has been revealed to be existed in embryonic liver and kidney as well as in the male mature germ cells and hematopoietic and lymphoid tissues [25]. Although the function of the *TNFAIP2* gene is still largely unknown, it has been suggested that *TNFAIP2* may play multiple roles in the development of organs, including vasculogenesis, blood cell differentiation, myelopoiesis and spermatogenesis. Furthermore, it was reported that the gene was repressed in marrow cells from acute promyelocytic leukemia (APL) patients and that its target mRNAs could be up-regulated by all-tans-RA (retinoic acid) [26].

MiR-184 is a single copy gene and evolutionarily conserved at the nucleotide level from flies to humans. Although its function remains unclear, some studies have suggested miR-184 as a potential oncogenic candidate. One study on squamous cell carcinoma (SCC) of the tongue showed that miR-184 might play a role in part in the anti-apoptosis and proliferation of the tongue SCC cells [27]. Another study firstly demonstrated that miR-184 significantly reduced tumor development and increased overall survival in an orthotopic murine model of neuroblastoma [28]. A later study found that miR-184 was involved in a common genetic pathway through targeting the serine/threonine kinase AKT2 by acting with MYCN transcription factor to inhibit neuroblastoma cell survival [29]. Taken together, these studies suggest a possible role of miR-184 in modulating cancer risk. However, it is not clear whether SNPs in its binding sites may further alter such modulation.

In our previous study in 1,077 patients with squamous cell carcinoma of the head and neck (SCCHN) and 1,073 cancer-free controls in a non-Hispanic white population, which evaluated the associations of the SNPs of *TNFAIP 2* gene with SCCHN risk [24], we also found that, compared with the rs8126 TT genotype, the variant CT and CC genotypes were associated with increased SCCHN risk in an allele-dose response manner [18]. In the genotype-phenotype correlation analysis of 37 SCCHN cell lines and peripheral blood mononuclear cells (PBMCs) from 43 SCCHN patients, the rs8126CC genotype was associated with reduced expression of *TNFAIP2* mRNA. Taken together, these findings suggest that the miR-184 binding site SNP (rs8126T>C) in the 3′UTR of *TNFAIP2* is functional, possibly by modulating *TNFAIP2* expression and contributes to SCCHN susceptibility [18]. Our findings in gastric cancer are consistent with those in the head and neck cancer.

Although our study did not reveal any main effect of other SNPs in the miRNA binding sites of *TNFAIP2* on overall risk of gastric cancer, we did find that the rs8126 CC variant homozygous genotype, compared with the combined genotypes (TC+TT), was associated with significantly increased cancer risk. The statistical evidence that could only be sustained in one subgroup of drinkers in the stratification analyses suggests the very limited sample size of the present study. Although tobacco use and alcoholic consumption have been regarded as major risks for gastric cancer, our matching design was intended to control for their confounding on the main effects of the selected SNPs, in which overmatching might happen due to possible associations between matching variables (age and sex) and the known risk factors (smoking and drinking) in this study population. In additional haplotype analysis, while four common frequency haplotypes failed to show any statistical cancer risk association, the minor frequency haplotype rs8126/rs1052823/rs710100: C-G-G proved to have increased risk, compared with the common haplotype T-G-G. The analysis of low-frequency haplotypes suggests a different conclusion, namely, that there may be an untyped low frequency SNP that is associated with gastric cancer. Additional studies are needed to validate this result.

However, since the present study is, to our knowledge, the first study on the *TNFAIP2* SNPs and gastric cancer risk, our findings are best considered preliminary, and larger studies are warranted to further assess the role of these SNPs in the etiology of gastric cancer. Another limitation in the current study was the lack of information on the *H. pylori* infection status of the subjects. Since *H. pylori* infection in gastric patients was relatively uncommon in the US [30], compared with those in South America [31] and Asian countries [32], patients recruited in our study were not tested for the infection upon their visit to the hospital. Therefore, inclusion of *H. pylori* information should be considered in future larger studies.
